# Gene Expression Regulation by Upstream Open Reading Frames and Human Disease

**DOI:** 10.1371/journal.pgen.1003529

**Published:** 2013-08-08

**Authors:** Cristina Barbosa, Isabel Peixeiro, Luísa Romão

**Affiliations:** 1 Departamento de Genética Humana, Instituto Nacional de Saúde Dr. Ricardo Jorge, Lisboa, Portugal; 2 Center for Biodiversity, Functional and Integrative Genomics, Faculdade de Ciências, Universidade de Lisboa, Lisboa, Portugal; University College London, United Kingdom

## Abstract

Upstream open reading frames (uORFs) are major gene expression regulatory elements. In many eukaryotic mRNAs, one or more uORFs precede the initiation codon of the main coding region. Indeed, several studies have revealed that almost half of human transcripts present uORFs. Very interesting examples have shown that these uORFs can impact gene expression of the downstream main ORF by triggering mRNA decay or by regulating translation. Also, evidence from recent genetic and bioinformatic studies implicates disturbed uORF-mediated translational control in the etiology of many human diseases, including malignancies, metabolic or neurologic disorders, and inherited syndromes. In this review, we will briefly present the mechanisms through which uORFs regulate gene expression and how they can impact on the organism's response to different cell stress conditions. Then, we will emphasize the importance of these structures by illustrating, with specific examples, how disturbed uORF-mediated translational control can be involved in the etiology of human diseases, giving special importance to genotype-phenotype correlations. Identifying and studying more cases of uORF-altering mutations will help us to understand and establish genotype-phenotype associations, leading to advancements in diagnosis, prognosis, and treatment of many human disorders.

## Introduction

Regulation of gene expression at the post-transcriptional level is increasingly being recognized as a key mechanism by which cells and organisms can rapidly change their gene expression patterns in response to internal or external stimuli. Emerging examples illustrate that expression of all genes is regulated at multiple post-transcriptional steps including mRNA processing, nuclear export and localization, stability, and translation of mature mRNA molecules. Translation itself is regulated by a diverse collection of mechanisms that act at the initiation step, as well as during elongation and termination and even after termination. Translational regulation at the initiation step can be mediated *via* different *cis*-acting elements present in the RNA 5′ leader sequence of specific transcripts; these elements include the secondary structure that is able to inhibit AUG initiation codon recognition due to a blockage of the scanning ribosome, internal ribosome entry sites (IRESs) that stimulate cap-independent translation, protein binding sites that either repress or promote translation in response to relaying molecular signals, non-AUG initiation codons, the AUG sequence context that affects efficiency of AUG recognition, and upstream AUG codons (uAUGs), in some cases, associated with upstream open reading frames (uORFs). uORFs are sequences defined by an initiation codon in frame with a termination codon located upstream or downstream of the main AUG. uORFs correlate with significantly reduced protein expression levels because they reduce the efficiency of translation initiation of the main downstream ORF in unstressed conditions [1], [2], or trigger mRNA decay [3]–[5]. However, in response to cellular stress, the presence of uORFs can promote the increased expression of certain stress-related mRNAs [6]. Nevertheless, there are other mRNAs for which it has been shown that some or all uORFs have no effect on translation [7], [8]. Indeed, from the published data, it is apparent that there are different mechanisms, some of them uORF(s) independent, which can be used by individual uORF-containing mRNAs to control protein synthesis.

Bioinformatic studies have now shown that about 49% of the human transcriptome contains uORFs, which are mostly conserved among species, suggesting evolutionary selection of functional uORFs [2], [9]–[12]. For example, genes as diverse as *CD36*, *MDM2*, *ERBB2*, *SOC1*, and *RARB* have conserved and experimentally characterized uORFs that regulate translation [10]. uORFs are conspicuously common in certain classes of mRNAs, including two-thirds of oncogenes and many other transcripts that encode proteins involved in important cellular processes, such as differentiation, cell cycle, and stress response [1], [6], [13]–[15]. As stated above, it has been suggested that uORFs are negatively correlated with protein production [2], [16], but until now, functional activity has been demonstrated for only a limited number of uORFs. Indeed, uORF-mediated translational regulation has been validated experimentally for about 100 eukaryotic transcripts, including around 30 human transcripts [2]. In addition, recent studies have described several transcripts where changes in the 5′ leader sequence that disrupt or create a uORF are associated with the development of human disease or disease susceptibility, revealing the importance of these *cis*-acting elements in gene expression regulation [2]. Bearing in mind the unequivocal examples already described, it is expected that uORF mutations may be involved in the genetic architecture of a wide variety of diseases, including malignancies, metabolic or neurologic disorders, and inherited syndromes.

In this review, we will briefly present the mechanisms through which uORFs are thought to regulate gene expression and how they can impact on the organism's response to different external conditions. Then, we will emphasize the importance of these structures in translational regulation by illustrating, with specific examples, how disturbed uORF-mediated translational control can be involved in the etiology of human disease, paying special attention to genotype-phenotype correlations. Identifying and studying more cases of uORF-altering mutations will help to establish and understand genotype-phenotype associations, leading to advances in diagnosis, prognosis, and treatment of many human disorders.

## uORFs as Translational Regulatory Elements

The process of mRNA translation can be divided into four stages—initiation, elongation, termination, and ribosome recycling—each of which requires a particular set of conditions and factors. Translation initiation is the rate-limiting step and, in eukaryotic cells, requires the participation of several eukaryotic initiation factors (eIFs) [17]. Canonical translation initiation is mediated by the recruitment of the cap-binding protein complex, namely eukaryotic initiation factor 4F (eIF4F), which comprises eIF4E, eIF4A, and eIF4G, to the mRNA 5′ end [18]. eIF4G has a binding site for eIF4E and the poly(A)-binding protein, which in turn is bound to the poly(A) tail, resulting in mRNA circularization [18]. The unwinding of the 5′ leader sequence by the ATP-dependent helicase eIF4A enables binding of the 40S ribosomal subunit. The association of eIF1, eIF1A, and eIF3 to the 40S subunit facilitates the binding of the ternary complex eIF2-GTP-Met-tRNAi [18]. The resulting 43S preinitiation complex can land next to the cap and scans in a 5′ to 3′ direction until it recognizes an AUG codon base pairing with Met-tRNAi [18], [19]. Upon recognition of the start codon, eIF5 stimulates GTP hydrolysis, resulting in the release of eIF2-GDP and probably other 40S-bound initiation factors. eIF5B catalyzes the recruitment of the 60S subunit to form an 80S ribosome, and elongation can start [18], [20].

Initially, it was assumed that the scanning 43S preinitiation complex would generally initiate translation at the first AUG codon encountered. However, several studies have shown that an AUG is not always recognized and that there are several factors that can influence this recognition, such as the sequence context of the AUG codon or the presence of strong secondary structures [21]. Indeed, it has been demonstrated that there are specific nucleotides surrounding the AUG codon whose presence correlates well with the strength of its recognition. The most efficient context for ribosome recognition and initiation of translation is known as the Kozak consensus sequence (GCCA/GCC**AUG** G). The nucleotides at positions −3 and +4 (underlined) are the most important ones for the definition of the context strength [22]. In the presence of a weaker context sequence, a mechanism called leaky scanning can occur, where the ribosome can either read the AUG codon or pass by it initiating translation at a downstream initiation codon [23].

**Figure 1:**
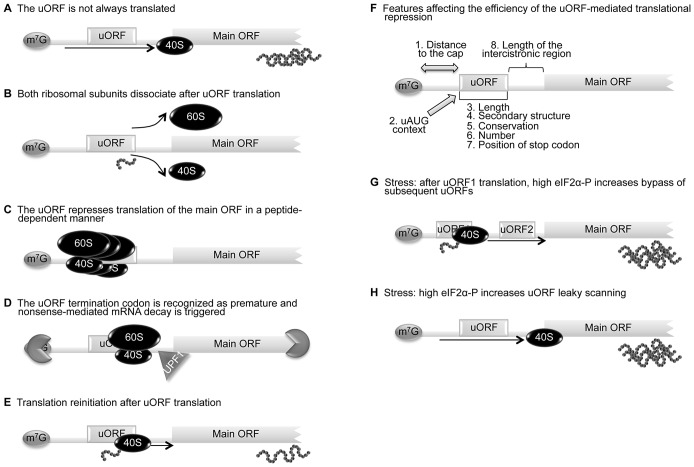
uORF-mediated translational control can occur through different mechanisms. (**A**) The leaky scanning mechanism is dependent on the efficiency of uAUG recognition; sometimes the ribosome can translate the uORF, but other times the scanning machinery bypasses the uAUG, recognizing the downstream AUG and translating the main ORF. (**B**) When a scanning ribosome recognizes and translates a functional uORF, there is synthesis of a small peptide; if translation termination of the uORF is efficient, both 60S and 40S ribosomal subunits might dissociate from the transcript and the main ORF is not translated. (**C**) A uORF can repress translation of the main ORF in a peptide-dependent manner; in this case, the uORF-encoded peptide interacts with the translating machinery and promotes ribosome blockage. (**D**) The termination codon of a uORF can be recognized as premature and nonsense-mediated mRNA decay (NMD) is triggered through a mechanism involving the UPF1 protein and ribonucleases. (**E**) After translation termination of the uORF, the 40S ribosomal subunit can remain associated with the transcript, resume scanning, and recognize the downstream main AUG—a mechanism designated as translation reinitiation. (**F**) The impact that the uORFs can have on translation depends on (i) distance between the 5′ cap (m^7^G) and the uORF (distance to the cap), (ii) context in which the uORF AUG is located (AUG context), (iii) length of the uORF, (iv) number of uORFs per transcript, (v) secondary structure of the uORF, (vi) conservation among species, (vii) length of the intercistronic sequence(s), and (viii) position of the uORF termination codon, upstream or downstream of the main initiation codon (length, number, secondary structure, conservation, position of stop codon). The increase of translational repression exerted by a uORF correlates with increasing distance between the m^7^G and the uORF, increasing length of the uORF and intercistronic sequence, a higher number of uORFs, and a stronger uAUG Kozak context. (**G**) In response to stress conditions, the presence of more than one uORF in a transcript can promote an increase in translation efficiency of the main ORF; the reinitiation after translation of the uORF1 is less efficient since there is less ternary complex available. Consequently, reinitiation will take more time/distance to occur and the ternary complex will only be available by the time the 40S ribosomal subunit has already bypassed the subsequent uORFs, augmenting the recognition of the main AUG. (**H**) In response to stress conditions, the presence of one uORF in a transcript can promote an increase of the corresponding protein levels; the higher levels of phosphorylated eIF2α contribute to increase leaky scanning of the uORF and translation of the main ORF is favored.

For a uORF to function as a translational regulatory element, its initiation codon must be recognized, at least at certain times, by the scanning 40S ribosomal subunit and associated initiation factors. When uORF recognition is regulated by the so-called leaky-scanning mechanism, ribosomes either scan through the upstream AUG codon (Figure 1A) or recognize it, initiating translation. In the case that the uORF is recognized by a scanning ribosome, the following alternative fates are available to the ribosome: (i) translate the uORF and dissociate (Figure 1B); (ii) translate the uORF and stall during either the elongation or termination phase of translation, creating a blockage to additional ribosomes (Figure 1C) or inducing mRNA decay (Figure 1D); or (iii) translate the uORF and remain associated with the mRNA, continue scanning, and reinitiate further downstream at either a proximal or distal AUG codon (Figure 1E). Translation reinitiation is thought to be an inefficient mechanism that happens only after translation of a short ORF [24]. Indeed, reinitiation is dependent on (i) the time required for the uORF translation, which is determined by the relative length of the uORF and the translation elongation rate; and (ii) the translation initiation factors involved in the translation initiation event [23], [25]. Several initiation factors need to remain associated with the ribosome during translation and even after the termination event so that reinitiation can occur [26], [27]. In this way, a ribosome that translates a shorter uORF (or with a higher translation rate) is more likely to reinitiate translation [25]. A key factor for translation reinitiation is the reacquisition of a new ternary complex (eIF2-GTP-Met-tRNAi); this complex is essential for the recognition of a downstream AUG by the scanning 40S subunit [28]. In fact, many studies have reported that longer intercistronic regions are more favorable for reinitiation, while for shorter ones the scanning time may not be sufficient for reacquisition of the ternary complex and the downstream AUG will therefore not be recognized [26], [27], [29]. The basis for the mechanism of translation reinitiation has not been completely elucidated. Therefore, it is essential to define more precisely which initiation factors promote reinitiation competence, as well as potential changes in the ribosomes that may be involved in this process.

As already stated, an additional feature of uORFs is their capacity to block the translational machinery in a peptide-dependent manner [30]; this might result in the stalling of other ribosomes that access the transcript, thereby dramatically decreasing the translation of the main ORF [31]. Examples of uORFs that function in a sequence-dependent manner are the receptor-like protein-tyrosine phosphatase J (*PTPRJ*) [32], the β2-adrenergic receptor, and the S-adenosylmethionine decarboxylase (*AdoMetDC*) [33]. The few examples described in mammals make it difficult to identify the conserved peptide sequences responsible, and identification of further uORFs with this ability is only possible experimentally. One study comparing full-length cDNA sequences from different plant species aiming to identify conserved peptide uORF sequences found that uORFs rich in serine, threonine, and/or tyrosine were present in nine homologous groups [34]. These amino acids are potential targets for phosphorylation that could possibly promote or inhibit ribosome stalling or translation initiation at downstream ORFs. Nevertheless, further characterization of this type of uORF is necessary before a consensus sequence can be annotated.

Despite the obvious complexity of uORF-mediated translational regulation, results from several studies have revealed that the impact the uORFs can have on translation depends on several variables, such as (i) the distance between the 5′ cap and the uORF, (ii) the context in which the uORF AUG is located, (iii) the length of the uORF, (iv) the secondary structure of the uORF, (v) conservation among species, (vi) the number of uORFs per transcript, (vii) the position of the uORF termination codon, upstream or downstream of the main initiation codon, and (viii) the length of the intercistronic sequence(s) (Figure 1F). Although all types of uORF can reduce protein expression in unstressed cells, four uORF properties are associated with greater translational inhibition; these are: strong uAUG context, evolutionary conservation, increased distance from the cap, and multiple uORFs in the 5′ leader sequence [2]. These properties reflect the impact that uORF(s) have in translational efficiency of the main ORF, when they are translated.

It is still unclear whether uORF-encoded peptides can play additional roles in the cell. Conceivably, uORF-encoded peptides could act both as translational regulators of the main ORF and as *trans*-acting factors in the cell. Further characterization of conserved uORFs might help to resolve this hypothesis.

## uORFs and mRNA Decay

The similarity between the cistronic organization of uORF-containing mRNAs to that of mRNAs containing a nonsense mutation has suggested the potential of the former to trigger the nonsense-mediated decay (NMD) pathway. NMD is one of the better characterized quality control mechanisms which acts as an mRNA surveillance pathway by degrading transcripts harboring premature translation termination codons (PTCs) [35]. However, in the last decade, several studies have also implicated NMD in the regulation of steady-state levels of physiological mRNAs, and many examples of natural NMD targets are indeed transcripts containing uORFs [3]–[5], [36], in which the uORF termination codon can be recognized as premature. The major challenge for this translation-dependent mechanism is to discriminate between a premature and a normal termination codon. This discrimination occurs when the ribosome is poised at the termination codon. According to current models, normal translation termination involves the interaction of the eukaryotic release factor 3 (eRF3) with the poly(A) binding protein cytoplasmic 1 (PABPC1) at the terminating ribosome, which stimulates a proper and efficient translation termination event [37]–[39]. However, if the termination codon location within a certain messenger ribonucleoprotein (mRNP) context does not allow PABPC1 to interact with eRF3, the terminating ribosome will stall, allowing its interaction with the NMD effector UPF1 and triggering NMD [40]. The “unified model” for NMD proposes that there are several features in the mRNP that can trigger the NMD response. For example, PTCs located at a greater distance from the poly(A) tail, as is the case for mRNAs harboring long 3′UTRs, can elicit NMD due to PABPC1 failing to interact with the termination complex [40]–[43]. Another NMD-triggering feature is the presence of at least one exon-exon junction more than 50 nucleotides downstream of the termination codon [44]. During splicing, the exon junctions are marked with a dynamic multiprotein complex designated exon-junction complex (EJC) that associates with the NMD factors UPF2 and UPF3 [45]. The presence of an EJC downstream of a termination codon allows the interplay between UPF1 at the terminating ribosome and UPF2 and/or UPF3, which results in UPF1 phosphorylation, irreversibly triggering NMD [46]. Consequently, PTCs located far, in a linear sense, from the poly(A) tail and associated PABPC1, in mRNAs containing residual downstream EJCs, are expected to elicit NMD [40]–[43]. Nevertheless, we have reported that AUG-proximal nonsense-mutated mRNAs evade NMD [47]–[50]. In such cases, there is establishment of an efficient translation termination event because of the ability of PABPC1 to travel with the ribosome, due to interactions with eIF4G and eIF3. This allows a repositioning of the PABPC1/eIF4G/eIF3 protein complex in the vicinity of the PTC at the translation termination event, blunting the NMD response and eliciting efficient termination [51]. Because the PABPC1/eIF4G/eIF3 complex might still be bound to the ribosome when it reaches the stop codon of a small ORF, eIF3 is in a favored position to promote reinitiation competence; as these interactions might be disrupted after some steps of translation elongation, transcripts carrying smaller ORFs are more competent for translation reinitiation than those with larger uORFs.

The termination codon of a uORF can be recognized as a PTC since it is distant from the 3′UTR signals and the corresponding transcript usually presents downstream EJCs located in the coding sequence of the main ORF [21], [52]. Examples of human transcripts whose uORFs trigger NMD are the interferon-related developmental regulator 1 (*IFRD1*) [53], the cystic fibrosis transmembrane conductance regulator (*CFTR*) [54], and *SMG5* [5]. However, some naturally occurring uORF-containing transcripts escape NMD. Indeed, uORFs often mediate translational repression of the protein coding ORF without an associated decrease in mRNA levels [21], [52]. The length of the uORF and the time taken to translate it are characteristics that influence the triggering of NMD (our unpublished data). According to our model [43], only transcripts harboring at least one uORF with a critical length would trigger NMD, while those with smaller uORF(s) could be NMD-resistant because of PABPC1 proximity to the uORF termination codon due to mRNA circularization during translation [50], [51]. In mammalian cells, the minimum size of the uORF that triggers NMD has been difficult to determine [3]; however, in plants, 35 codons is the threshold [55]: transcripts with longer uORFs are NMD-sensitive and those with shorter uORFs are NMD-resistant. Also, in plants, increasing the reinitiation predisposition has no effect on NMD, which contradicts the notion that reinitiation would prevent the destabilization of the mRNA [55]. Nevertheless, in mammalian cells, some transcripts with long uORFs, which are NMD-targets under normal circumstances, become resistant to NMD during stress conditions, depending on the phosphorylation of eIF2α [53], [56]. *IFRD1* is a documented example of a uORF with 52 codons that responds to the phosphorylation of eIF2α by increasing mRNA stability [53]. One possible explanation for NMD inhibition in response to eIF2α phosphorylation is that under these conditions, leaky scanning through the uORF increases and thus the corresponding stop codon is not recognized, which impairs NMD. This example illustrates how complex and puzzling the inhibitory effect of a uORF and the response to stress conditions can be. In any case, these data demonstrate that cells have evolved different mechanisms that contribute to the integrated stress response, among which inhibition of NMD also contributes to increased expression of stress-response proteins.

## uORFs and the Cellular Response to Stress Conditions

Translational regulation mechanisms are able to mediate rapid and reversible changes in protein expression as a cellular response to internal and external stimuli. One of the most commonly used mechanisms for inhibiting global translation is by phosphorylation of the initiation factor eIF2 [57]. In order to be recycled, eIF2 is recharged with GTP by the guanine nucleotide exchange factor (GEF) eIF2B. However, when eIF2 is phosphorylated on serine 51 of its α subunit, it becomes a competitive inhibitor of eIF2B, preventing eIF2 recycling and reducing translation initiation rates by lowering the ternary complex concentration [57]. In mammalian cells, phosphorylation of eIF2α on serine 51 is a major mechanism that regulates initiation of translation in response to various cellular stresses, including virus infection, nutrient deprivation, iron deficiency, and accumulation of unfolded proteins in the endoplasmic reticulum (ER) [57]. Depending on the specific cellular stress, eIF2α is phosphorylated by at least four different kinases, including double-stranded RNA-activated kinase (PKR), general control non-derepressible 2 kinase (GCN2), heme-regulated inhibitor kinase (HRI), and PKR-like ER kinase (PERK). Following stress-induced eIF2α phosphorylation, translation of normal cellular mRNAs is repressed, while the translational initiation of selected mRNAs involved in stress response is stimulated [57].

A second mechanism for nonspecifically reducing levels of protein synthesis involves interfering with m^7^G cap recognition, thereby preventing recruitment of the translational machinery to the mRNA [58]. The m^7^G cap is recognized by eIF4E as part of the eIF4F complex; however, there are several eIF4E-binding proteins (4E-BPs) which compete with eIF4G for a binding site on eIF4E and prevent eIF4F complex formation [59]. The strength of binding of 4E-BPs to eIF4E is controlled by phosphorylation: hypophosphorylated 4E-BPs bind strongly, while phosphorylated 4E-BPs bind weakly.

**Figure 2:**
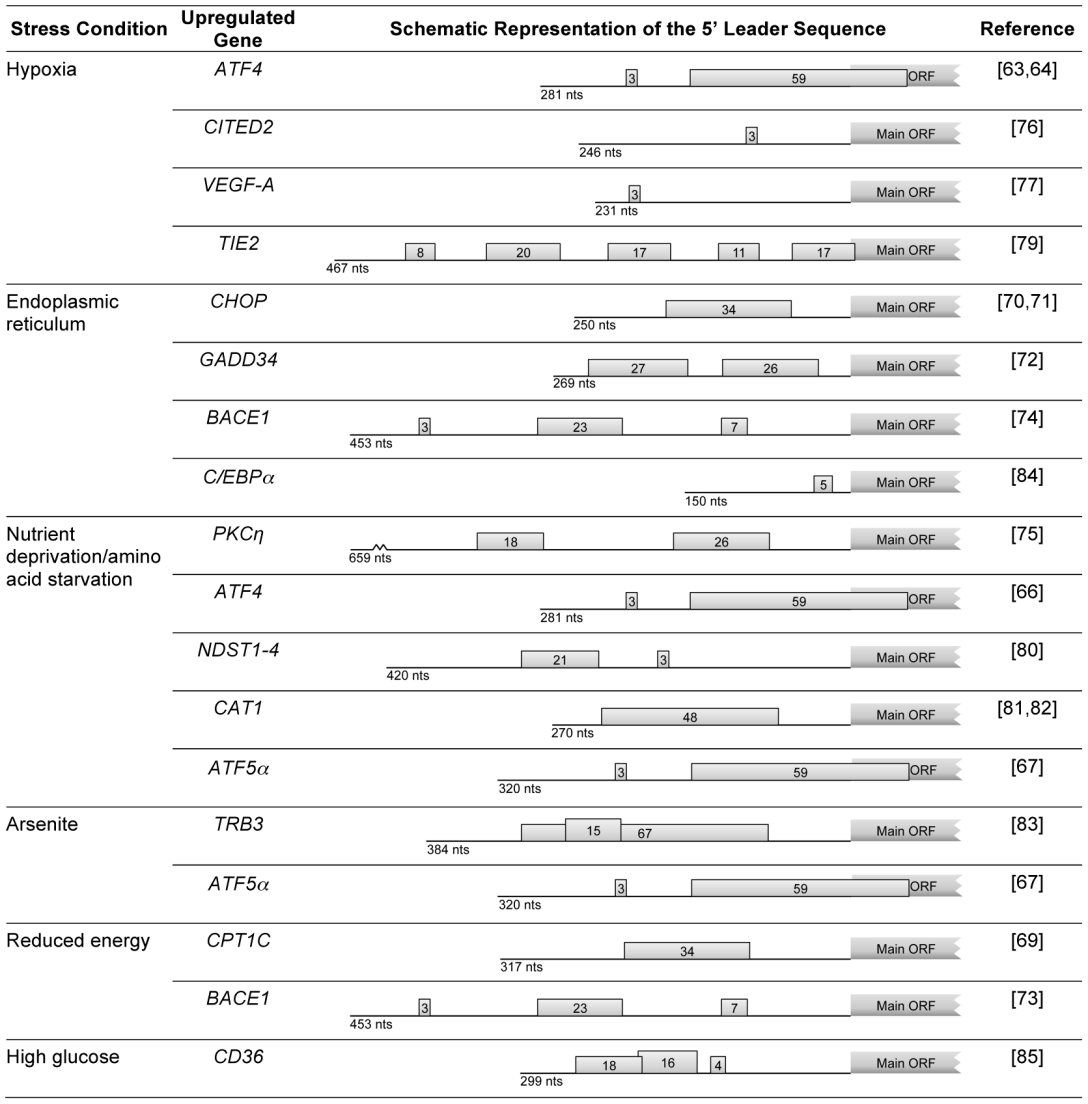
Examples of human genes encoding mRNAs that, under stress conditions, evade global repression of translation and are upregulated due to the presence of uORFs. For each mRNA, the schematic representation of the 5′ leader sequence is shown with the length (in nucleotides; nts) indicated below each representation; boxes with numbers represent the uORF(s), where the number indicates the corresponding length in codons.

As stated above, accumulating evidence has revealed that in response to abnormal stimuli, general translation is inhibited. However, alternative mechanisms of translation initiation and translational control act to maintain the synthesis of certain proteins required either for the stress response or to aid recovery from stress. These pathways are evolutionarily conserved and have been shown to significantly impact translation in organisms as diverse as yeast and humans. In many cases, features in the 5′ leader sequence of the corresponding mRNAs, such as IRESs and regulatory uORFs, are important for them to evade global repression of translation. For example, when eIF2 is phosphorylated and consequently global translation is inhibited, the presence of uORF(s) in a transcript can promote an increase in the corresponding protein levels (Figure 1G and Figure 1H). The yeast transcription factor *GCN4* is one of the better studied examples of a transcript containing uORFs that are able to respond to cell stress. This transcript harbors four uORFs in its 5′ leader sequence. The first of the four uORFs is always efficiently translated regardless of the nutritional conditions. In unperturbed cells, rapid reloading of ribosomes and initiation cofactors allows translation of uORFs 2–4 while inhibiting the translation of the main ORF. In conditions of amino acid starvation, reinitiation after translation of the uORF1 is less efficient since there is less ternary complex available. Consequently, reinitiation will take more time/distance to occur and the ternary complex will only be available by the time the 40S ribosomal subunit has already bypassed the subsequent uORFs, thereby augmenting the recognition of the main AUG [60]. This mechanism allows a fast response to nutritional stress [61], [62]. The stress response gene that encodes the activating transcription factor 4 (*ATF4*) is the prototypical mammalian example of this type of regulation [63]. ATF4 promotes transcriptional upregulation of specific target genes in response to cellular stress. *ATF4* expression at the translational level is regulated by two uORFs, with the second overlapping the AUG of the *ATF4* coding sequence, although in a different reading frame (Figure 2). Under normal conditions, when eIF2α is not phosphorylated and ternary complex is not limiting, the scanning preinitiation complex recognizes the first uORF and translates a short peptide, and the 60S ribosome dissociates upon reaching the stop codon marking the end of the uORF. The 40S ribosomal subunit that remains associated with the mRNA is then able to recruit ternary complex and initiate translation of the second uORF. Because the second uORF overlaps with the main coding sequence, this prevents translation of the *ATF4* coding sequence. However, in conditions of reduced ternary complex availability, initiation of the second uORF is less likely, as there is less chance of the scanning ribosomal subunit recruiting the ternary complex required for start codon recognition [63], [64] (Figure 2). By this mechanism, a reduction in active eIF2 induces increased protein expression from mRNAs carrying the correct arrangement of uORFs (Figure 1G and Figure 1H) [65], [66]. This is also the case for the human ATF5 [67]; like ATF4, ATF5 is a transcription factor of the cAMP-response element binding protein (CREB)/ATF family, which is encoded by two transcripts (*ATF5α* and *ATF5β*) with alternative 5′ leader sequences [68]. The 5′ leader sequences of *ATF4* and *ATF5α* have similar configurations and both contain two conserved uORFs [64], [66]–[68] (Figure 2). Similar to what occurs in the *ATF4* mRNA, the *ATF5α* uORFs are involved in protecting cells from amino acid limitation, as well as from arsenite-induced oxidative stress, through phosphorylation of eIF2α [67]. Interestingly, the regulatory mechanisms governing variable ATF4 and ATF5 expression in response to eIF2α phosphorylation, under different conditions of stress, are likely due to a combined effect of translational and transcriptional control of *ATF4* and *ATF5* mRNAs. In addition, global cellular adaptation to stress includes the transcriptional upregulation of ATF4 and ATF5 targets. Nevertheless, other genes activated by eIF2α phosphorylation may also function in conjunction with ATF4 and ATF5, as well as their targets.

As stated, genes with uORFs in their transcripts are good candidates to be upregulated in response to eIF2α phosphorylation. An example of regulated expression *via* uORF(s) is the carnitine palmitoyltransferase 1C (*CPT1C*) gene (Figure 2). CPT1C regulates metabolism in the brain in situations of energy surplus. The presence of a uORF in the 5′ leader sequence represses the expression of the main ORF. However, this repression is relieved in response to specific stress stimuli like glucose deprivation and palmitate-BSA treatment [69]. The mRNAs that encode the CCAAT/enhancer-binding protein homologous protein (*CHOP*) [70], [71], growth arrest DNA-inducible gene 34 (*GADD34*) [72], and β-site APP (amyloid precursor protein) cleavage enzyme 1 (*BACE1*) [73], [74] are also examples where the phosphorylation of eIF2α is responsible for the translational derepression (Figure 2). The majority of these transcripts bear more than one uORF, resulting in an effect similar to the one seen in *GCN4*, *ATF4*, or *ATF5α* (see above). Although it seems that transcripts with only one uORF can also be regulated by this mechanism as is the case for the *CHOP* transcript, the underlying molecular basis for this remains poorly understood. Chen et al. have reported that in cells under anisomycin treatment, uORF-mediated *CHOP* translation is controlled by the dissociation of phosphorylated eIF4E from 4E-BP. A key finding of this study is that the phosphorylation of both eIF4E and eIF2α is crucial for *CHOP* stress-responsive translational regulation [71]. These authors also showed that anisomycin activates both Mnks and mTOR signaling pathways which converge at eIF4E for *CHOP* uORF-mediated translation, in addition to phosphorylated eIF2α [71]. Despite the fact that many questions still need to be answered, these two pathways have been implicated in the induction of translation of uORF-containing transcripts, such as protein kinase C [75], *ATF4* [66] in response to amino acid starvation, *CITED2* [76] in response to hypoxia, or *CPT1C* [69] in response to specific stress stimuli, namely glucose deprivation and palmitate-BSA treatment.

In addition, vascular endothelial growth factor A (*VEGF-A*) [77], *p27* [78], endothelial cell tyrosine kinase receptor (*TIE2*) [79], N-deacetylase/N-sulfotransferase (*NDST*) [80], and cationic amino acid transporter 1 (*CAT1*) [81], [82] provide other examples of transcripts regulated by functional uORFs (Figure 2); however, it is interesting to note that in these cases, uORFs are located within an IRES, which is translated through a cap-independent mechanism. In the case of *CAT1* mRNA, it has been demonstrated that induction of IRES activity requires the translation of the uORF located within the IRES [82]. The translation of the uORF unfolds an inhibitory structure in the mRNA 5′ leader sequence, creating an active IRES through RNA-RNA interactions between the 5′ end of the leader sequence and downstream sequences, which increases CAT1 protein synthesis [82].

There are other interesting examples of how *cis*-acting elements and different gene expression mechanisms can act together for a specific outcome [83]–[85] (Figure 2). In the case of the tribbles homolog 3 (*TRB3*) gene, in response to arsenite exposure, there is binding of ATF4 to the promoter which leads to a switch in promoter usage; this results in the production of a transcript with no uORF, while under normal conditions two transcripts are produced: one with a uORF in the 5′ leader sequence and one with no uORF [83]. For the *C/EBPα* gene, 2-cyano-3,12-dioxooleana-1,9-dien-28-oic acid (CDDO) augments C/EBPα activity in acute myeloid leukemia cells by translationally enhancing the p42/p30 *C/EBPα* isoform ratio in a *C/EBPα* uORF-dependent manner [84]. In another case, high glucose conditions increase *CD36* mRNA translational efficiency that results in increased expression of the macrophage scavenger receptor CD36, due to ribosomal reinitiation following translation of a uORF. Increased translation of the macrophage *CD36* transcript provides a mechanism for accelerated atherosclerosis in diabetics [85].

A final example is the *HER2* oncogene that encodes a 185 kDa transmembrane receptor tyrosine kinase. HER2 overexpression occurs in numerous primary human tumors and contributes to 25–30% of breast and ovarian carcinomas. Synthesis of HER2 is controlled in part by a uORF that represses translation of the downstream main coding region. HER2 overexpression in cancer cells seems to be due to an interaction of 3′UTR with the uORF through an RNA-binding protein, thus overriding translational inhibition mediated by the *HER2* uORF [86]. Even though the precise mechanism by which this interaction occurs is still unknown, it provides further evidence of how uORFs and other gene expression pathways can act together for the modulation of the expression of regulatory genes and of the individual phenotype. In addition, the examples shown here suggest that the translational control mediated by uORFs may involve several steps of mRNA metabolism, may include unfolding of mRNA structures, specific sequences, or *trans*-acting factors, may occur in a context-dependent manner, and may respond differently to stress-activated translation initiation factors.

## uORFs and Human Disease

Given that uORFs reduce translational efficiency, it is clear that polymorphisms or mutations that create, disrupt, or modify uORFs are likely to affect protein expression and may impact individual phenotypes. Indeed, when Calvo and colleagues searched for uORF-altering variants within 12 million single nucleotide polymorphisms (SNPs) in the human dbSNP database [2], [87], they identified uORFs created or deleted by a polymorphism in 509 genes; 366 of these genes encode transcripts harboring multiple uORFs, whereas the remaining 143 mRNAs have a single uORF [2]. This study also showed that these uORFs induce a 30–60% decrease in protein levels when compared to the protein levels expressed from the corresponding allele without the uORF-altering SNP variant [2]. As a concrete example, an SNP was described that alters the human clotting factor XII (*FXII*) 5′ leader sequence, and has been associated with several thromboembolic conditions due to differences in circulating FXII plasma levels [88]. This SNP consists of a common C to T polymorphism with prevalence of the T allele estimated at 20% in Caucasian and 70% in Asian populations [89], [90]. It is located at position −4 of the *FXII* 5′ leader sequence (where the A of the main AUG start codon is nucleotide +1), introduces a very short uORF (with 2 codons), and simultaneously alters the AUG Kozak sequence context of the factor *FXII* coding sequence. Kanaji and colleagues have experimentally confirmed that the T allele does not affect mRNA levels, but reduces protein levels by about 50%, increasing the predisposition to thrombosis [90]. More recently, it was demonstrated that this protein reduction is indeed due to the presence of the 2-codon uORF, while the disruption of the Kozak consensus sequence is not responsible for the observed variation in human FXII protein levels [2] (Table 1). This example shows how SNPs, found through genetic analyses in the 5′ leader sequence of transcripts, cannot be disregarded, as even if they do not affect mRNA levels they can affect protein levels and be associated with human disease. This region should, therefore, be systematically explored when investigating the molecular mechanism of a disease.

**Table 1:** Examples of human diseases associated with polymorphisms or mutations that introduce/eliminate uORFs or modify the encoded uORF peptide.

Disease	Gene	Mode of Pathogenesis	Reference
**Polymorphisms/mutations that create uORFs**			
1. Thrombotic predisposition	*FXII*	The -4C to T polymorphism creates a uORF that reduces mRNA translation efficiency from the main ORF(a)	[2], [88]–[90]
2. β-Thalassemia	*HBB*	The -29G to A mutation creates a new translation initiation codon in a favorable Kozak consensus sequence, which leads to the introduction of a new uORF that overlaps with the main ORF, but out of frame, and decreases translation efficiency from the main ORF(a)	[2], [91]
3. Carney complex type 1	*PRKAR1A*	The -97G to A mutation creates a uORF that overlaps with the main ORF, but out of frame, and decreases translation efficiency from the main ORF(a)	[2]
4. Van der Woude syndrome	*IRF6*	The -48A to T mutation creates a uORF that overlaps with the main ORF, but out of frame, and decreases translation efficiency from the main ORF(a)	[2], [92]
5. Gonadal dysgenesis	*SRY*	The -75G to A mutation creates a second uORF and reduces mRNA translation efficiency from the main ORF(a)	[2], [93]
6. Hereditary pancreatitis	*SPINK1*	The -53C to T mutation creates a uORF and reduces mRNA translation efficiency from the main ORF(a)	[2], [94]
7. Melanoma predisposition	*CDKN2A*	Both described -21C to T and -34G to T mutations create a uORF that reduces mRNA translation efficiency from the main ORF(a)	[95], [96]
8. Familial hypercholesterolemia	*LDLR*	A single C nucleotide deletion (at position -22) creates a uORF and reduces mRNA translation efficiency from the main ORF(b)	[97]
9. Disseminated bronchiectasis	*CFTR*	The -34C to T mutation creates a uORF overlapping, but out of frame, with the CFTR protein coding sequence, which decreases gene expression by reducing mRNA stability and translation efficiency from the main ORF(a)	[98]
10. Congenital hyperinsulinism	*KCNJ11*	The -54C to T mutation creates a new translation initiation codon in a favorable Kozak consensus sequence, which leads to the introduction of a new uORF that overlaps with the main ORF, but out of frame, and decreases translation efficiency from the main ORF(b)	[99]
11. Rhizomelic chondrodysplasia punctata	*PEX7*	The -45C to T mutation creates a new translation initiation codon in a favorable Kozak consensus sequence, which leads to the introduction of a new uORF that overlaps with the main ORF, but out of frame, and decreases translation efficiency from the main ORF(b)	[100]
12. Proopiomelanocortin deficiency	*POMC*	The -11C to A mutation creates a new translation initiation codon in a favorable Kozak consensus sequence, which leads to the introduction of a new uORF that overlaps with the main ORF, but out of frame, and decreases translation efficiency from the main ORF(b)	[101]
13. Levodopa responsive dystonia	GCH1	The -22C to T mutation creates a new translation initiation codon that leads to the introduction of a new uORF overlapping with the main ORF, but out of frame, and decreases translation efficiency from the main ORF(b)	[102]
14. Juvenile hemochromatosis	*HAMP*	The -25G to A mutation creates a new translation initiation codon, which leads to the introduction of a new uORF overlapping with the physiological ORF, but out of frame, and decreases translation efficiency from the main ORF(a)	[103]
**Polymorphisms/mutations that disrupt uORFs**			
15. Marie Unna hereditary hypotrichosis	*HR*	The -321A to G mutation disrupts one of the existing uORFs and results in an increased translational efficiency of the main *HR* physiological ORF(a)	[104], [105]
16. Thrombocythemia	*TPO*	-31G to T mutation generates a new stop codon in uORF 7 and thereby shortens uORF 7 by 42 nucleotides. The truncated uORF 7 no longer extends past the physiological initiation codon, and thus it improves translational efficiency by allowing translation reinitiation(a)	[106]–[109]
		The G to C transversion in the splice donor site of intron 3 of the *TPO* gene leads to mRNAs with shortened 5′ leader sequence that are more efficiently translated than the normal *TPO* transcripts because they lack uORF 7, which normally inhibits translation; a novel N-terminus is created by fusion of uORF 5 with the *TPO* coding sequence(a)	[110]
		A single G nucleotide deletion (at position -50) in the 5′ leader sequence of the *TPO* gene causes a frameshift in the 5′ leader sequence of *TPO* mRNA that places uORF 7 in frame with the *TPO* coding sequence, neutralizing the strong inhibitory effect of uORF 7 and creating a novel N-terminus for the TPO protein(a)	[111]
**Polymorphisms/mutations that modify the encoded uORF peptide**			
17. Schizophrenia predisposition	*DRD3*	The -204A to G polymorphism within a 36-codon uORF originates a Lys9Glu amino acid substitution in the uORF-encoded peptide that might decrease efficiency of ribosomal blockage; this change causes an increase in the DRD3 protein levels(b)	[112]
18. Aspirin-exacerbated respiratory disease	*WDR46*	The -36G to A polymorphism originates a Gly18Arg amino acid substitution in the uORF-encoded peptide(b)	[113]
19. Arrhythmogenic right ventricular cardiomyopathy	*TGFβ3*	The -30G to A mutation within an 88-codon uORF originates a Arg36His amino acid substitution in a putative 88–amino acid inhibitory peptide encoded by the uORF; this change causes an increase in the TGF-β3 protein levels(a)	[114]
20. Bipolar affective disorder and major depression	*HT3A*	-42C to T mutation originates a Pro16Ser amino acid substitution in the uORF-encoded peptide and is postulated to decrease the efficiency of the uORF repression causing an increase in the HT3A protein levels(a)	[115]
**Other alterations**			
21. Acute myeloid leukemia	*C/EBPα*	The *C/EBPα* uORF modulates the expression ratio of three N-terminally distinct protein isoforms that are translated from subsequent in frame initiation codons within the *C/EBPα* transcript; an increase in expression of the shorter isoform is associated with acute myeloid leukemia(a)	[116]
22. Breast cancer	*C/EBPβ*	The *C/EBPβ* uORF modulates the expression ratio of three N-terminally distinct protein isoforms that are translated from subsequent in frame initiation codons within the *C/EBPβ* transcript; an increase in expression of the shorter isoform due to the inactivation of the uORF is associated with breast cancer(a)	[116], [117]
23. Several tumors	*MDM2*	A switch in promoter usage favors transcription of an isoform without uORFs which overexpresses MDM2 protein in comparison with what occurs in normal cells, where one isoform with two uORFs is mainly expressed(a)	[119]
24. Alzheimer's disease	*BACE1*	Elevated levels of phosphorylated eIF2α induce a bypass of the inhibitory mechanism exerted by *BACE1* uORFs, which leads to enhanced BACE1 expression(a)	[120], [121]

Position of the mutation is relative to the main AUG start codon, where the A is nucleotide +1.

(a) It has been experimentally tested to affect translational efficiency.

(b) It is not experimentally tested.

In addition to polymorphisms that can affect uORFs, rare mutations that create or disrupt uORFs may also cause disease, as has been shown for several human genes [2], [91]–[115] (Table 1). Indeed, several mutations that eliminate or create uORFs that alter protein levels have been associated with human disease. Calvo and colleagues have experimentally demonstrated, in five genes (*HBB*, *PRKAR1A*, *IRF6*, *SRY*, and *SPINK1*), that mutations that create a uORF decrease protein expression levels to 30%, or less, of those from the normal allele, and these reduced protein levels are responsible for the associated disease phenotype [2]. Notably, with the *SRY* and *SPINK1* genes, the mutation creates a second uORF within the 5′ leader sequence. Thus, the strong suppression of protein expression by these mutations offers a simple mechanistic basis for their pathogenicity [2]. Another study has shown that predisposition to melanoma can be caused by mutations that introduce a uORF into the 5′ leader sequence of the mRNA encoding the cyclin-dependent kinase inhibitor protein (*CDKN2A*) [95], [96]. Other examples of human diseases associated with mutations that create a uORF include familial hypercholesterolemia (low-density lipoprotein receptor gene; *LDLR*) [97], cystic fibrosis (*CFTR*) [98], congenital hyperinsulinism (potassium inwardly-rectifying channel, subfamily J, member 11; *KCNJ11*) [99], rhizomelic chondrodysplasia punctata (peroxisomal biogenesis factor 7; *PEX7*) [100], proopiomelanocortin deficiency syndrome (proopiomelanocortin; *POMC*) [101], levodopa-responsive dystonia (guanosine triphosphate cyclohydrolase I; *GCH1*) [102], and juvenile hemochromatosis (hepcidin; HAMP) [103] (Table 1). Although the majority of the polymorphisms/mutations referred to here that create a uORF have been experimentally tested for their influence on translation, in the case of *LDLR*, *KCNJ11*, *PEX7*, *POMC*, and *GCH1* mRNAs, further studies are needed to confirm the effect of the corresponding mutation on translational efficiency (Table 1).

Contrary to the effect of mutations that create a uORF, the repression exerted by a functional uORF can be modulated by mutations, or alternative processing of the transcript, that disrupt the uORF, thus influencing the translational rate of the main ORF. In either case, there is a change in organism homeostasis that affects individual phenotype. An illustration of a genetic alteration that disrupts a uORF is a mutation described in the initiation codon of an inhibitory 34-codon uORF located in the 5′ leader sequence of the mRNA that encodes the human hairless homolog (*HR*) protein. This mutation has been associated with the symptomatic condition of Marie Unna hereditary hypotrichosis, which is a rare autosomal dominant form of genetic hair loss [104], [105]. Functional analysis showed that this mutation results in increased translation of the main *HR* physiological ORF [104], [105]. Another noteworthy example is the thrombopoietin (*TPO*) gene [106]. Translation of *TPO* mRNA is physiologically strongly inhibited by the presence of seven uORFs in its 5′ leader sequence. Directed mutagenesis of all uAUGs in the *TPO* mRNA restores translational efficiency, demonstrating that translational inhibition of TPO biosynthesis is entirely mediated by uORFs [106]. The uORF defined by the seventh uAUG was shown to exert the strongest negative effect on translation. This uAUG is in a good Kozak consensus context and the uORF extends beyond the physiological start site, thus preventing reinitiation [106]. Mutations in the 5′ leader sequence of the *TPO* gene, which cause hereditary thrombocytosis, inactivate the inhibitory function of uORF 7 and abolish this translational control [106]–[111]. In these cases, pathologically high TPO levels are observed, leading to an increased number of platelets in the peripheral blood and increased thrombosis risk. One particular mutation was demonstrated to introduce a translation termination codon in the 5′ leader sequence in frame with uORF 7. As the new in frame stop codon produces a uORF entirely located in 5′ leader sequence, it confers the ability to reinitiate at the main ORF. This new regulation mechanism by uORF 7 produces a weaker translational repression, causing an increase of the TPO protein levels [107]–[109]. In another case, a point mutation (G to C transversion) in the +1 position of the splice donor site of intron 3 causes exon skipping and results in loss of exon 3 that normally encodes a large part of the 5′ leader sequence. As a consequence, the mutant *TPO* mRNA lacks uORF 7, which normally inhibits translation, and encodes a novel N-terminus created by fusion of uORF 5 with the *TPO* coding sequence [110]. A different mutation consists of a single G nucleotide deletion in the 5′ leader sequence of the *TPO* gene that causes a frameshift in the 5′ leader sequence of *TPO* mRNA, which places uORF 7 in frame with the *TPO* coding sequence, neutralizing the strong inhibitory effect of uORF 7 and creating a novel N-terminus for the TPO protein [111]. These data clearly illustrate how TPO expression is tightly regulated at the translational level.

As mentioned above, uORFs may differ in their efficiency and in the mechanisms by which they exert translational repression of the main ORF. In some cases uORFs repress translation because the corresponding encoded peptide is able to promote a blockage in the translating ribosome [30]. Consequently, specific nucleotide substitutions that alter the uORF coding sequence and originate an amino acid substitution might affect the efficiency of ribosomal blockage and thus protein expression from the main ORF. For example, amino acid substitutions that decrease efficiency of ribosomal blockage might decrease the translational repression exerted by the uORF, and therefore they might increase protein levels, which might lead to clinical manifestations. This is the case for the human dopamine D3 receptor (*DRD3*) gene [112]. Sivagnanasundaram and colleagues have screened for polymorphisms to assess their contribution to the association of DRD3 with schizophrenia. Their data have shown that one of the SNPs found in the 5′ leader sequence encodes a change of one amino acid residue from lysine to glutamic acid within a 36-codon uORF, which correlates to an increased schizophrenia predisposition [112] (Table 1). Another example is the G to A transition described in the *WDR46* gene that originates an amino acid change from glycine to arginine at codon 18 of a uORF in the *WDR46* transcript; this variant is associated with higher risk of aspirin-exacerbated respiratory disease [113] (Table 1). In a different study, authors identified the transforming growth factor-β3 (*TGFβ3*) gene as being involved in arrhythmogenic right ventricular cardiomyopathy, a progressive and genetically determined myocardial disease, due to a G to A transition in the *TGFβ3* 5′ leader sequence, which leads to an arginine to histidine substitution at codon 36 of a uORF with 88 codons; it has been experimentally proven that this change causes an increase in the *TGFβ3* protein levels [114] (Table 1). Moreover, the human *HT3A* mRNA, which encodes the subunit A of the type 3 receptor for 5-hydroxytryptamine (serotonin), contains two uORFs, in frame with the main ORF. A -42C to T mutation in the second uORF of *HT3A* is associated with bipolar affective disorder and major depression; it has been experimentally shown that this mutation increases translation efficiency of the 5-HT3A subunit [115] (Table 1). For these pathologies, elucidating the mechanisms through which uORFs can affect downstream translational efficiency, depending on the amino acid sequence of the uORF-encoded peptide, may constitute a tool for the development of new and more effective drug treatments.

Another intriguing regulatory function of uORFs is observed in transcripts harboring alternative downstream initiation codons within their main ORF. This is exemplified by CCAAT/enhancer binding protein β and α (*C/EBPβ* and *C/EBPα*, respectively), in which uORFs control the expression ratio of functionally distinct protein isoforms by sensing the translational status of the cell [116]. Recently, interesting work using C/EBP uORF mice has corroborated the role of uORFs in pathophysiology (Table 1). This genetic mouse model has provided the proof-of-principle for the physiological relevance of uORF-mediated translational control in mammals [116], [117], as targeted disruption of the uORF initiation codon within the *C/EBPβ* mRNA resulted in deregulated C/EBPβ protein isoform expression, associated with defective liver regeneration and impaired osteoclast differentiation [116], [117].

Another fascinating regulatory function of uORFs occurs in transcripts encoded by genes with cryptic promoters—e.g., the oncoprotein MDM2, which is overexpressed in a number of human tumors, particularly in osteosarcomas [118]. This overexpression can result from a change in mRNA structure due to a switch in promoter usage. There are two transcripts from the *MDM2* gene that differ only in their 5′ leader sequence: a long form (*L-MDM2*) that carries two uORFs and a short form (*S-MDM2*) without uORFs. In these tumors, the switch in promoter usage yields enhanced cellular levels of the *S-MDM2* mRNA isoform, which is efficiently translated. On the contrary, the *L-MDM2* mRNA is less efficiently transcribed and its translation is repressed by two functional uORFs [119]. Overall, MDM2 becomes overexpressed in tumors due to the preferential transcription of the *S-MDM2* isoform that is not under translational regulation (Table 1) [119]. This set of data illustrates how disrupted uORF-mediated translational regulation can affect expression levels of oncogenes or tumor suppressor genes, and thus contribute to the pathophysiology of many forms of cancer.

As previously discussed, uORF-mediated translational regulation has the ability to respond to stress conditions, which is a feature that can also be associated with human disease. This may be the case for the beta-site amyloid precursor protein-cleaving enzyme 1 (*BACE1*) gene, which encodes an enzyme involved in the production of beta-amyloid plaques in the brain of patients with Alzheimer's disease (AD). The enhanced production of this enzyme occurs without corresponding changes in *BACE1* mRNA levels and seems to occur at the translational level. The complex *BACE1* 5′ leader sequence contains three uORFs preceding the *BACE1* initiation codon that might be involved in the enhanced production of this enzyme characteristic of humans with AD. It has been hypothesized that aging and other factors such as cardiovascular disease or traumatic brain injury might impair brain energy metabolism that leads to a higher phosphorylation of eIF2α. Indeed, it has been shown that energy deprivation induces phosphorylation of the eIF2α, which increases the translation of *BACE1* mRNA [73]. Under these conditions, the BACE1 protein levels might increase due to a uORF(s)-mediated translational derepression, leading to beta-amyloid overproduction, which could be an early, initiating molecular mechanism in sporadic AD (Table 1) [7], [8], [73], [74], [120], [121]. However, some other data is consistent with the hypothesis that the translation efficiency of the *BACE1* initiation codon may be increased in patients with AD by molecular mechanisms that enhance shunting or increase the relative accessibility of the *BACE1* initiation codon, without the involvement of uORF(s) [7].

Although phosphorylation of eIF2α in response to cellular stress has been unequivocally shown to increase *BACE1* translation [73], [74], the involvement of uORF(s) in the stress-dependent mechanism of translation initiation is more controversial [7], [8], [120], [121]. Indeed, it has been shown that the *BACE1* uORF(s) have little or no effect on BACE1 expression in unstressed cells [7], [8]. Instead, it may be the GC-rich region of the *BACE1* 5′UTR that forms a constitutive translation barrier, which could prevent the ribosomes from efficiently translating the *BACE1* mRNA [8]. The exact role of the three *BACE1* uORFs in its translational regulation needs further evaluation.

In the examples discussed here, all the uORF-altering polymorphisms/mutations have been reported in the literature as demonstrating segregation with the disease. However, some of them, although present within a gene known to underlie the disease when disrupted, were not followed up experimentally (by using reporter assays) to confirm their impact on translational efficiency (Table 1). In any case, these examples highlight the importance of searching for uORF changes—in addition to coding alterations—underlying disease and draw attention to the need for recognition of these structures as potential therapeutic targets.

The recent advances in next-generation sequencing technologies certainly represent a quantum leap toward (i) the identification of a large number of novel disease-associated uORF alterations, (ii) the subsequent uncovering of predictive genotype-phenotype correlations in many areas of human pathology, and (iii) the recognition of uORFs as possible therapeutic targets.

## Conclusions

It is currently accepted that uORFs may control protein expression through the involvement of different mechanisms. On the other hand, emerging data has been showing how uORF-mediated translational control can affect cell fate decisions. Although only a limited number of described uORF alterations have been associated with human disease, it is now clear that such alterations can be involved in the pathophysiology of different disorders and in modulating the severity of the individual phenotype. However, it is our belief that the approaches used to date have yet to reveal all the mechanisms of translational control by uORFs. Consequently, further characterization of the mechanisms through which altered uORFs might be associated with human disease will be of great value in the discovery of novel diagnosis and prognosis biomarkers as well as therapeutic targets, thereby allowing for the development of new control strategies for many diseases, including malignancies, metabolic or neurologic disorders, and inherited syndromes. In addition, the knowledge gathered from this type of research (namely on the role of uORFs in the response to external and internal stimuli) will certainly contribute to a better understanding of the complex network of interactions leading to homeostasis maintenance and health.
